# Development and Evaluation of Automated Tools for Auditory-Brainstem and Middle-Auditory Evoked Potentials Waves Detection and Annotation

**DOI:** 10.3390/brainsci12121675

**Published:** 2022-12-06

**Authors:** Ourania Manta, Michail Sarafidis, Nikolaos Vasileiou, Winfried Schlee, Christos Consoulas, Dimitris Kikidis, Evgenia Vassou, George K. Matsopoulos, Dimitrios D. Koutsouris

**Affiliations:** 1 Biomedical Engineering Laboratory, School of Electrical and Computer Engineering, National Technical University of Athens, 15780 Athens, Greece; 2 Department of Psychiatry and Psychotherapy, University of Regensburg, 93053 Regensburg, Germany; 3 Laboratory of Experimental Physiology, National and Kapodistrian University of Athens, 11527 Athens, Greece; 4 1st Department of Otorhinolaryngology, Head and Neck Surgery, National and Kapodistrian University of Athens, Hippocrateion General Hospital, 15772 Athens, Greece

**Keywords:** auditory evoked potential (AEP), auditory brainstem response (ABR), auditory middle latency response (AMLR), automated wave-annotation, waveforms

## Abstract

Auditory evoked potentials (AEPs) are brain-derived electrical signals, following an auditory stimulus, utilised to examine any obstructions along the brain neural-pathways and to diagnose hearing impairment. The clinical evaluation of AEPs is based on the measurements of the latencies and amplitudes of waves of interest; hence, their identification is a prerequisite for AEP analysis. This process has proven to be complex, as it requires relevant clinical experience, and the existing software for this purpose has little practical use. The aim of this study was the development of two automated annotation tools for ABR (auditory brainstem response)- and AMLR (auditory middle latency response)-tests. After the acquisition of 1046 raw waveforms, appropriate pre-processing and implementation of a four-stage development process were performed, to define the appropriate logical conditions and steps for each algorithm. The tools’ detection and annotation results, regarding the waves of interest, were then compared to the clinicians’ manual annotation, achieving match rates of at least 93.86%, 98.51%, and 91.51% respectively, for the three ABR-waves of interest, and 93.21%, 92.25%, 83.35%, and 79.27%, respectively, for the four AMLR-waves. The application of such tools in AEP analysis is expected to assist towards an easier interpretation of these signals.

## 1. Introduction

An auditory evoked potential (AEP) is an electrical signal elicited by the brain when a time-locked auditory stimulus is presented [1,2]. The final AEP signal consists of the averaged responses to thousands of stimulus repetitions [3]. AEPs are a type of non-invasive and non-behavioural test, and their primary benefits are their simplicity, objectivity, reproducibility, and cost-effectiveness [4]. Based on an individual’s AEP response, audiologists are able to examine possible obstructions along the neural pathways running to the brain. Moreover, this signal may be useful for ruling out or confirming hearing impairment, especially in neonates, and for medicolegal reasons in order to exclude benign tumours of the acoustic nerves, such as acoustic neuromas [5]. 

AEPs are categorised as early (auditory brainstem responses—ABRs), middle (auditory middle latency responses—AMLRs), and late (auditory late latency responses—ALLRs), depending on their time of occurrence following the triggering event [6]. 

**Figure 1:**
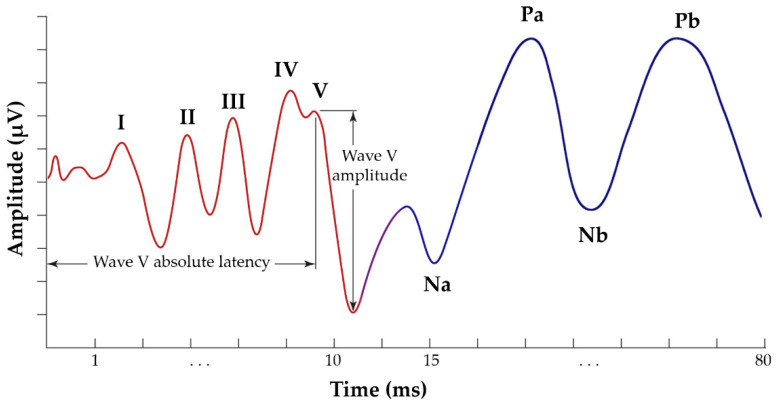
Typical annotated ABR signal, presenting the five waves of interest, from I to V (red waveform) and AMLR signal, presenting the four waves of interest, Na, Pa, Nb and Pb (blue waveform).

In particular, an ABR is a series of acoustically stimulated signals that reflect synchronised neuronal activity along the neural pathways. It is considered one of the most reliable electrophysiological techniques, with a long history of application [7,8]. More specifically, within 10 ms of the onset of a moderate-intensity click stimulus, the derived ABR consists of a sequence of five peaks, originating from the auditory nerve and brainstem. The majority of researchers mark peaks using capital Roman numerals (I through V) (Figure 1). Wave I of the ABR reflects the activity of spiral ganglion cells in the distal eighth-auditory-nerve, wave II originates from the globular cells in the cochlear nucleus, wave III is generated by the cochlear-nucleus spherical cells and globular cells, and waves IV and V are generated by the medial superior olive and its projections to the nuclei in the lateral lemniscus and the inferior colliculus [9,10]. Typically, the amplitude of these electrophysiological responses is less than one microvolt (μV) [5,11]. 

The AMLR may be captured using the same equipment as the ABR. AMLR is generally recorded during a temporal frame of 80 to 100 ms, occurring between approximately 12 and 60 ms after the external stimulation. It is believed that the thalamus and auditory cortex are the generators of this response. It is defined as a waveform consisting of four waves of interest: two troughs (Na and Nb), and two peaks (Pa and Pb) (Figure 1). AMLR is a potential that is sensitive to low frequencies, and the discrepancy between the behavioural auditory-threshold and the electrophysiological threshold is roughly 10 dB [12,13], despite the scant data available. In addition, it is worth noting that there is considerable physiological variability in the morphology of these waveforms, with the Nb and Pb components being present inconsistently among normal individuals. These factors may also explain why “normative” data for AMLRs is scarce [5,14]. Hence, it is evident that the establishment of criteria for AMLRs annotation is a complicated endeavour, as it must also account for the existence of abnormal patterns.

ALLR is generated by non-primary cortical regions and is used to assess the integrity of the auditory system beyond the level of middle latency response. It usually occurs between 60 and 800 ms after the external stimulus [15].

AEPs have predictable patterns, and consist of distinct waves (peaks and troughs) which are actually the signal’s main turning points and are generated by certain stations along the acoustic pathway [4]. The latencies (the time between the initial auditory stimulus and the peak or trough of a waveform [8]), absolute amplitudes, and inter-wave intervals [16] corresponding to the signal’s waves are the primary measurements of an AEP. When analysing and interpreting these waveforms, clinicians consider these measurements as metrics. Consequently, the initial stage in the analysis of an AEP is the identification of the waveform’s peaks and troughs. Nevertheless, the detection and characterization of the waveforms still require manual annotation by clinicians, as the present software, accompanied by recording systems, is frequently unable to choose the proper waves. The identification of these waves is a demanding task, which requires sufficient clinical experience, and, therefore, many recording systems have been developed, including relevant embedded annotation tools. However, their use in clinical practice is limited. 

The available tool for AEP-waves annotation is usually the software which accompanies the commercially available systems through which the recordings are obtained. The Eclipse recording system (EP15 and EP25 modules) [17] of the Interacoustics A/S company, the ABR screening system SmartEP [18] of the Intelligent Hearing System Corporation, the MP system [19] of Biopac Systems Inc., the EP-PreAmp [20] of Brain Products, and the OAE screening system NEURO-AUDIO [21] of Neurosoft are among the most well-known platforms that capture, monitor, and interpret AEP waveforms within their proprietary software. Regarding ABR waveforms, two individual attempts which aim at analysing them were identified: the sABR software [22] from the University of British Columbia, and Ωto_abR [23], a web application for the visualisation and analysis of click-evoked auditory brainstem responses. However, in both cases, the proposed annotation tools for ABR signals have not been validated by clinicians. Regarding AMLR waveforms, no individual attempts achieving automated annotation were identified.

In summary, there are many challenges associated with the annotation of ABR and AMLR waveforms. As mentioned above, the existing software for this purpose is of little use in practice. This fact dictates the need for manual annotation of these signals to be conducted only by clinicians with adequate clinical experience. In the case of AMLR waveforms, the lack of normative data and protocols for the recording setup, and the morphology of the signals, add difficulty to the overall task. Therefore, especially in this AEP subtype, the waves of interest are even more difficult to discern.

In the context of this study, two tools were developed to automatically identify and annotate the waves of interest that constitute the recorded ABR and AMLR signals. These automated annotation tools could facilitate and assist specialists from different fields to interpret AEP signals and, thus, to diagnose and treat patients.

## 2. Materials and Methods

### 2.1. Data Collection

In order to develop the automated annotation tools, we utilized recorded ABR and AMLR waveforms collected within the European project “UNITI” (Unification of treatments and Interventions for Tinnitus Patients) [24]. The waveforms were recorded and extracted using the Interacoustics Eclipse system (module EP25) [25]. This system offers the possibility of exporting the raw measurements of the recorded AEPs in *.xml* (Extensible Markup Language) files. The exported files did not contain any patient data. All the UNITI project’s waveforms were recorded heterochronously by clinicians from four different centres: Hippokratio General Hospital of Athens, Athens, Greece; Klinikum der Universitaet Regensburg, Regensburg, Germany; Charité—Universitaetsmedizin Berlin, Berlin, Germany; and Hospital Universitario San Cecilio, Granada, Spain. All centres used the same procedures regarding electrode placement and recording set-ups, which were pre-agreed by the clinicians of the UNITI consortium. This contributed to the uniformity in the structure and quality of the collected data, ensuring the ability of comparison and group analysis. Within the scope of this study, the two annotation tools were developed and validated using solely data from two centres (Hippokratio General Hospital of Athens, and Charité—Universitaetsmedizin Berlin).

### 2.2. Stimulus and Acquisition Parameters

The type of stimulus used for the recording of ABR waveforms was one click, and the repetition rate was 22 stimuli per second, at intensity levels of 80 and 90 dB nHL. The recorded signal was filtered with a high-pass filter set at 33 Hz, 6 dB/octave, and a low-pass filter set at 1500 Hz, and the sample rate was 30 kHz. For the recording of AMLR waveforms, the stimulus used was one 2 kHz tone-burst with a duration of 28 sine waves, presented at a rate of 6.1 Hz/s and at an intensity level of 70 dB nHL. The recorded signal was filtered with a high-pass filter set at 10 Hz, 12 dB/octave, and a low-pass filter set at 1500 Hz, and the sample rate was 3 kHz. More details on the settings used for the stimulus and on the acquisition parameters of each test can be found in Table 1 and Table 2.

### 2.3. Reading Raw Data

The appropriate pre-processing steps in order to reconstruct and visualise the waveforms of the two AEP subtypes from the raw data are outlined below. These procedures were performed before the development process of the two proposed annotation tools.

Initially, it was important to grasp the present ABR and AMLR datafiles’ contents. There were important parameters in the *.xml* files that had to be comprehended and utilised in order to reconstruct and visualise the ABR and AMLR signals. The *.xml* header description may be found in the “Additional Information” handbook for the EP25 module (https://www.manualslib.com/products/Interacoustics-Eclipse-Ep25-11647463.html, accessed on 4 December 2022).

All the pre-processing and analysis steps were conducted using the R programming language. The R programming language and its accompanying packages (e.g., *XML* [26], *xml2* [27], *ggplot2* [28], *signal* [29], *seewave* [30], *tuneR* [31], *gsignal* [32], *MIMSunit* [33], *base* [34]) were used to read the exported *.xml* files, extract the associated data measurements, rebuild and visualise the waveforms, and, subsequently, develop the two automated annotation tools. 

Initially, all waveforms (*.xml* files) from the two clinical centres were separated into distinct folders (one folder per AEP subtype). For each folder, the *list.files* function of the *base* package (version: 3.6.2) was used to select the *.xml* files and to create a character vector with all the *.xml* files’ names. In that way, we were able to call any *.xml* file conveniently. The data of each selected *.xml* file was read, and all the related *.xml* tags and attributes were stored in a list, via the *xmlParse* function of the *xml2* package (version: 1.3.3) and the *xmlToList* function of the *XML* package (version: 3.99-0.11). This made the extraction of useful data extremely simple, as each attribute of the ABR or AMLR waveform was also a field of the stored list.

The raw measurements were stored in two fields, IPSI_A_RAW and IPSI_B_RAW, in the *.xml* files. These fields correspond to the values stored in the two buffers present in the Eclipse system. The final waveform presented by the Eclipse software is obtained from the average of these two fields. Therefore, according to the suggestions of the Eclipse EP25 manual (in the “Research Module” section), we developed a function which generated the averaged signal and converted the raw values to μV. Regarding the matching between the time stamps and the signal samples, the Eclipse system is calibrated in order to adjust for the delay in the transducer in such a way that the first sample corresponds to the instant when the stimulus is completed. This is achieved in practice by starting the stimulus before the sampling starts. Thus, taking into account the ‘PrestimulusSamples’ and ‘SampleRate’ tags of the *.xml* file, the correct matching between each sample (amplitude value) and time stamp of occurrence was achieved.

### 2.4. Visual Display Filtering and Wave Detection

The extracted *.xml* data did not include the visual display filters (VDFs); however, clinicians normally apply them in order, to perform the annotation on the waveforms under interpretation and evaluation. Therefore, the next required step in order to reconstruct the waveforms was the application of these filters in accordance with the clinicians’ setup (Table 1 and Table 2). For this step, the R package *signal* (version 0.7-7), and, more specifically, the functions *fir1* (for low-pass filters) and *butter* (for high-pass filters) were used, with the proper arguments.

The process of detecting the signal’s waves of interest started by finding the local extrema of the waveforms. To find them, the *find_peaks* function (https://github.com/stas-g/findPeaks, accessed on 4 December 2022) was embedded [23]. This function identifies the local maxima of a number sequence, ignoring those that exist for a small subset of the sequence (which can be determined). More specifically, a data frame “x” is given as an input to the function, and a vector, which contains the index of the data frame corresponding to the local maxima, is returned as an output. For the detection of local minima, i.e., the troughs of the waveform, the opposite values “-x” are assigned to the function. Therefore, the function returns only the possible waves of interest and not all the existing waves which could emerge due to small fluctuations in AEP signals or noise at the electrodes.

### 2.5. Visualisation

Subsequent to the previous steps, the reconstruction and visualisation of each waveform was implemented with the R package *ggplot2* (version: 3.3.6). This package is widely used in R to create elegant and complex graphs, providing the ability to present unidimensional and multidimensional data without extensive analysis. 

### 2.6. Development Process of the Automated Annotation Tools

Initially, the two clinicians (authors CC and DK) performed a manual annotation of the waves of interest, independently. When the *.xml* files were exported from the Eclipse software, the labelled positions (defined as serial numbers of samples) corresponding to the waves of interest were included in the relevant “Jewetts” tags of the *.xml* files. The positions of the waves were mapped to time stamps in accordance with the procedure presented previously (Section 2.3), and this information was stored in data frames. Thus, it could be used as the “gold standard” during the construction and performance evaluation of the automated annotation tools.

Subsequently, some initial logical conditions were created for the identification of each wave of interest, based on the existing literature and clinicians’ annotations. These logical expressions were embedded in our annotation tools, so as for the waves that best meet the criteria could be selected. If no wave is found within the specified conditions, the value for the associated wave is set as NA, and our tool proceeds to search for the next wave of interest. The ABR annotation tool detects waves (peaks) I, II, III, IV, and V, and calculates and stores their corresponding amplitudes and latencies. The AMLR annotation tool finds the four waves of each signal (trough Na, peak Pa, trough Nb, and peak Pb), and calculates and stores their corresponding amplitudes and latencies.

**Figure 2:**
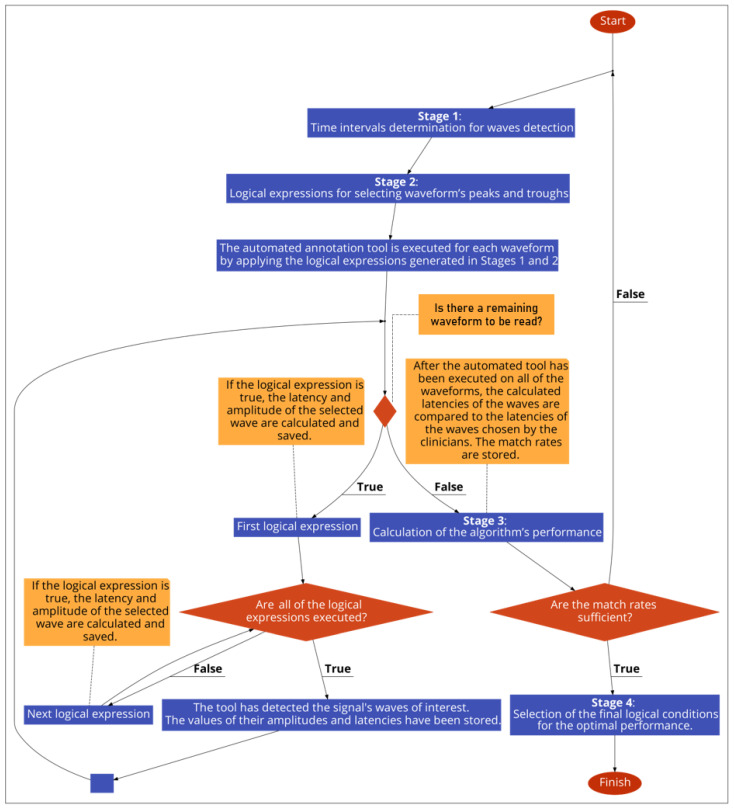
The development process of each automated annotation tool.

The development of the automated annotation tool of each AEP subtype was constituted by four stages (Figure 2):

1. Determination of appropriate time-intervals for each wave of interest under detection;
2. Development of logical (Boolean) expressions for selecting the waveform’s peaks and troughs;
3. Calculation of the algorithm’s performance, by comparing its selected latencies with the clinicians’ latencies, and optimisation of the logical conditions, by adjusting the parameters of stages 1 and 2. Iteration of stage 3, after each change in parameter of the earlier stages, and storing of the performance values in a data frame;
4. Selection of the final logical conditions that result in the optimal performance.

These stages were implemented for each of the two tools separately, and are described in detail hereinafter:

Stage 1: time-interval determination for wave detection.

Initially, the first step in order to develop the tools for the automated detection of the waves of interest, was to define the “normative” time intervals for each wave, based on current knowledge and the literature. Regarding ABR waveforms, the expected time of occurrence of the peak (latency) Jewett is roughly approximated to 1.5 ms for peak I, 3.5 ms for peak III, and 5.5 ms for peak V [5,16]. With regard to AMLR waveforms, the acceptable latency-ranges for the normative values are between 18.00–25.00 ms for the Na trough, 24.00–36.00 ms for the Pa peak, 34.00–47.00 ms for the Nb trough, and 55.00–80.00 ms for the Pb peak, according to [35]. According to Musiek [5,36] the Na trough is expected to occur within 12.00–21.00 ms, and the Pa peak within 21.00–38.00 ms, while no information was available for the Nb and Pb waves. According to Goldstein [5,37] who was the one who selected and established the labels Na, Pa, Nb, and Pb for the four waves of interest, the normative value for the Na trough is between 16.25–30.00 ms, for the Pa peak between 30.00–45.00 ms, for the Nb trough between 46.25–56.26 ms, while for the Pb peak, he did not specify an interval. At the same time, a literature review was performed for the interpeak latencies (i.e., between the peaks). It was found that, for ABR, the interpeak latencies between peaks I-II and III-V are expected to be 2.0 ms, and, consequently, the time distance from peak I to peak V is of the order of 4.0 ms [5]. Similarly, the time interval from the Na trough to the Pa peak is expected to be within 7.50–18.75 ms [5].

Notably, all the waveforms analysed in this study belonged to chronic-tinnitus patients, the majority of whom (86.69%) were also diagnosed with hearing loss. Therefore, it is possible and plausible that abnormalities in their AEP signals could exist. These abnormalities could include prolonged or shorter latencies than normally expected, or the absence of some waves of interest. Therefore, there was a need for adjustment of these intervals in order not to exclude possible waveform abnormalities. Regardless of the utilised data, we considered that the extension of normative intervals in such tools was essential, since the aim was to be able to detect and recognise any abnormalities in the waves. These aberrations can occur for many different reasons, such as medical (e.g., a disease, degree of hearing loss) or non-medical (e.g., head morphology, and stimulus and waveform acquisition-parameters). It is worth mentioning that the time-interval extension was performed gradually and conservatively, as there was no information in the literature about how much a wave interval can be extended and can deviate from its expected normal range of duration.

Stage 2: logical expressions for selecting waveform’s peaks and troughs.

The second stage was the creation of the logical expressions able to select which of the identified troughs and peaks constitute waves of interest of the signal. The creation of the conditions for each wave of interest is combinatorial and exclusive. This means that, in addition to the afore-mentioned time intervals, the maximum (from the peaks) or minimum (from the troughs) amplitudes were taken into consideration, in combination with the position of the previous and following waves of interest. Thus, the trough and peak amplitudes were searched within “reasonable” time intervals and within specific inter-peak/trough times, based on current knowledge and the literature.

It is important to note that in cases where the algorithm had to choose between more than one time stamp to select the wave of interest, the priority was given to time-related rather than amplitude-related conditions. In cases where there was more than one peak within a time interval, priority was given to the peak that was within the normal ranges and not the expanded ranges, unless the peak amplitude were much larger in the expanded range than in the normal range. Similarly, this procedure was repeated for the cases where there was more than one trough, where the minimum values were searched.

More specifically, in order to create the conditions for the ABRs, the search for the three main peaks was carried out sequentially, by locating firstly peak I, then peak III, and, finally, peak V. For these peaks, there was no overlap of time interval, by definition. Waves II and IV were also identified, even though they have not been used so far in clinical interpretation, so that they could be utilised as assisting waves for the annotation of peaks I, III, and V.

To create the conditions for the AMLRs, the search was initiated by locating the Pa peak (as the basic component of this AEP) and, after locating this wave, proceeding to the search for the earlier trough, Na, and the later trough, Nb, with the corresponding logical expressions for these components. Then, in accordance with the time stamps of Pa and Nb (if found), the algorithm proceeded to search for the Pb peak. Initially, priority was given to the maximum and minimum values of the amplitudes, within the respective time intervals, for the selection of the waveform’s troughs and peaks. In other words, the same procedure which was followed for detecting the waves of interest in the ABR waveforms was adopted. However, after multiple trials (at the performance-calculation stage), it was shown that better performances were achieved using combinatorial approaches. Specifically, the Pa peak was still chosen based on the maximum amplitude, within certain intervals, but for the other waves the time distance from the Pa peak was given greater importance than the absolute amplitude of the peaks and troughs.

Stage 3: calculation of the algorithm’s performance.

Following stages 1 and 2, each algorithm was embedded in a “for loop”, which read all waveforms of a specific AEP-subtype and stored the proposed algorithm’s selections for the waves of interest in a data frame. In the same data frame, the patient identifiers, the corresponding ear of each recording and the stimulus intensity, were also included. The algorithm’s selected waves of interest, which were based on the conditions of the first two stages, were compared with the clinicians’ selected waves. We made the assumption of accepting a proposed selection as “correct” when the deviation was up to ±4 samples from the selected waves by the clinicians. It should be noted that each recorded signal is composed of 450 samples in total, regardless of whether it was the ABR or AMLR signal. In addition, the signals for the ABR waveforms correspond to a total time-duration of 15 ms, so the time difference of the maximum deviation of the 4 samples corresponds to a time difference equal to 4*15/450 ≅ 0.13 ms. Similarly, the signals for the AMLR waveforms correspond to a total time-duration of 150 ms, so the time difference of the maximum deviation of the 4 samples corresponds to a difference equal to 4*150/450 ≅ 1.33 ms. The acceptance of a wave selection as “correct” with a deviation of up to 4 samples, was made in order to overtake any “wrong” clinicians’ wave selection, either due to possible “faulty” use of the cursor or an inability to locate the peak / trough through naked-eye observation.

Stage 4: selection of the final logical conditions for the optimal performance.

After comparing the algorithm’s performance (i.e., match rates), the waveforms in which any incorrect annotation was detected were revisited. This helped us to revise and enhance the conditions, and thus to optimise them after repeated changes and testing. Therefore, the third stage (performance-calculation) was repeated several times, on the entire set of waveforms. This means that after each change of conditions in either stage 1 or stage 2, stage 3 was repeated, and the match rates for the new algorithm’s proposed waves were saved. The match rates for each wave’s set of conditions were compared with each other, and the set of conditions that showed the higher match-rate was finally selected.

### 2.7. Determination of Presence of PAM Artefacts on the AMLR Waveforms

A very common phenomenon in AMLR-waveform recordings is that artefacts are captured in the recordings. The recording of artefacts results in distortion and alteration of the acquired AEP, and automatically renders the waveforms clinically “useless”. In the worst case, if the specialist is not able to identify the artefacts and proceed to the evaluation of the relevant signals, this may lead to a wrong interpretation and diagnosis. In AMLRs, the most commonly encountered artefact is that resulting from muscle interference, specifically of the mastoid muscle of the ear, also called the post-auricular-muscle (PAM) response artefact. The presence of the PAM artifact on an AMLR recording can seriously interfere with the identification of its waves of interest [38]. The main characteristic of this artefact is a sharp component spike in the range of 13 to 15 ms [39], (i.e., it always starts before the appearance of the basal (robust) Pa component [14]), with its amplitude being much larger than the amplitude of the Pa component. The PAM artefact is more likely to occur in tense patients with an ipsilateral inverted-electrode in the earlobe or mastoid, and at auditory-stimulus intensity levels > 70 dB nHL [39]. One solution suggested by physicians is for the patient to remain still and rest comfortably, with the head supported and the neck neither flexed nor extended. However, despite the physicians’ instructions and suggestions, PAM is still recorded in many instances. 

The second form of artefact that can occur in AMLRs is the artefact of filter settings and steep filter-slopes (with a filter bandwidth of 30 to 100 Hz and/or slopes of 24 to 48 dB/octave). However, these artefacts are easily eliminated using wide filter-settings and by extending the high-pass-filter setting downwards, to a lower frequency cut-off.

For these reasons, in the context of a more appropriate evaluation of the performance of the proposed AMLR annotation tool, we considered it necessary to re-conduct the evaluation performance, this time without the inclusion of the waveforms with the PAM artefact. To achieve this, all available AMLR waveforms were re-examined for the possible presence of PAM artefacts and, when present, the corresponding waveforms were removed.

## 3. Results

### 3.1. Data Collection

The data-collection stage led to the acquisition of a total of 1046 waveforms derived from 180 patients (94 patients from Hippokratio General Hospital of Athens and 86 patients from Charité—Universitaetsmedizin Berlin). The 698 waveforms (368 from Athens and 330 from Berlin) pertained to ABR signals, of which 359 were acquired with an acoustic stimulus of 80 dB intensity and 339 with a stimulus of 90 dB. The remaining 348 waveforms related to AMLR signals, with 186 of them acquired from Athens and 162 acquired from Berlin. 

### 3.2. Reading Raw Data

**Figure 3:**
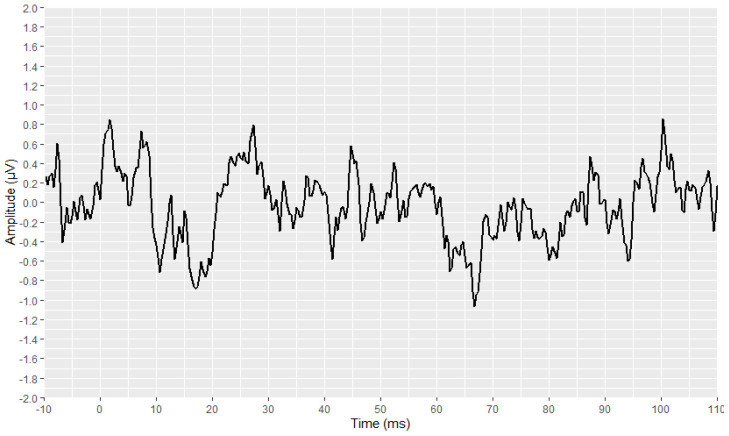
Visualisation of an AMLR waveform from raw data (in black).

**Figure 4:**
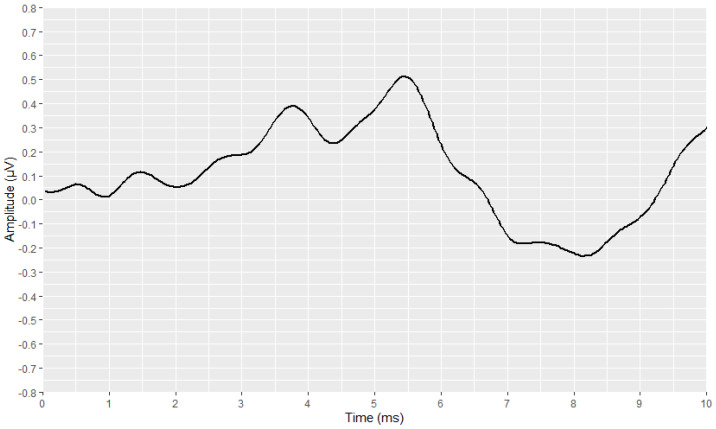
Visualisation of an ABR waveform from raw data (in black).

**Figure 5:**
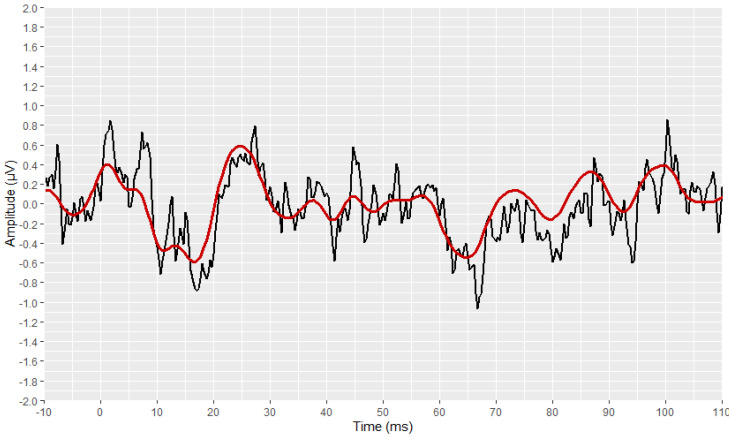
Visualization of an AMLR waveform after applying the visual display filters (raw signal is depicted in black and filtered signal is depicted in dark red).

**Figure 6:**
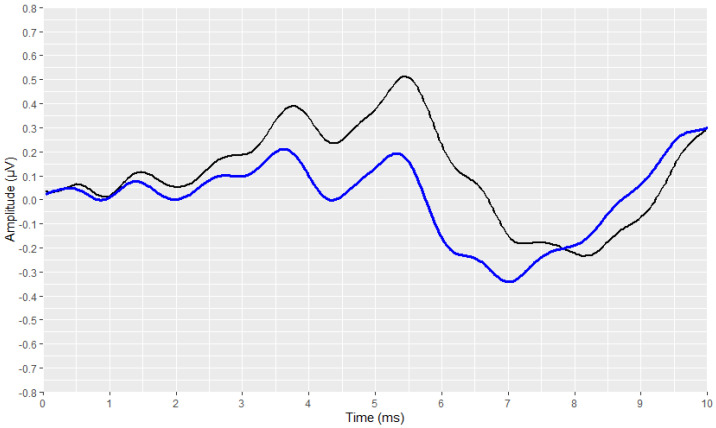
Visualization of an ABR waveform after applying the visual display filters (raw signal is depicted in black and filtered signal is depicted in blue).

**Figure 7:**
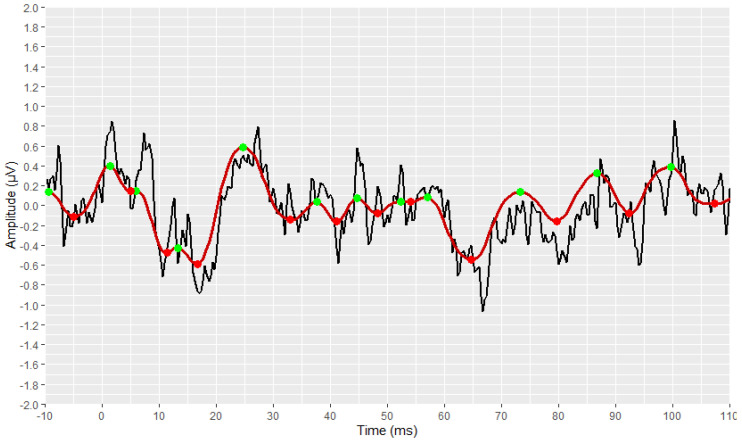
Detection of all local extremes (peaks in green, troughs in red) of the filtered AMLR signal (raw signal is depicted in black and filtered signal is depicted in dark red).

**Figure 8:**
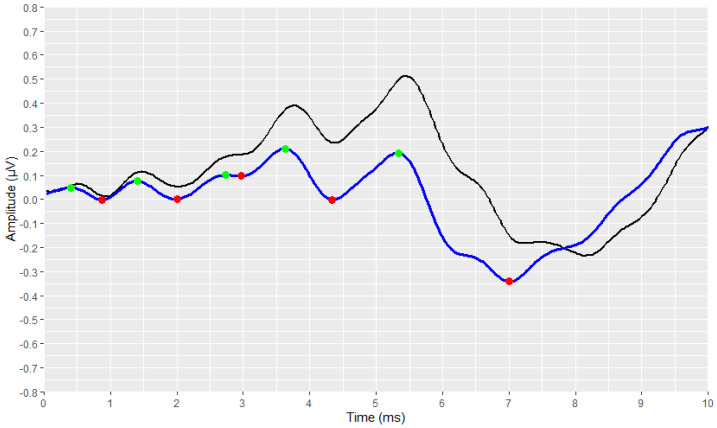
Detection of all local extremes (peaks in green, troughs in red) of the filtered ABR signal (raw signal is depicted in black and filtered signal is depicted in blue).

**Figure 9:**
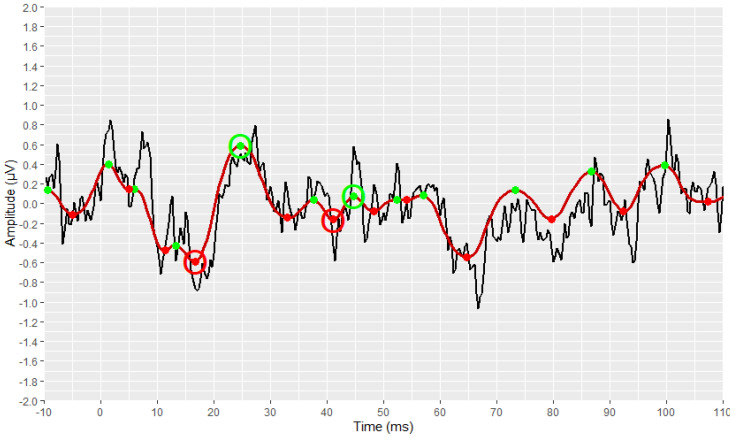
Detection of the AMLR signal’s waves of interest as resulting from the implementation of the AMLR automated annotation tool.

**Figure 10:**
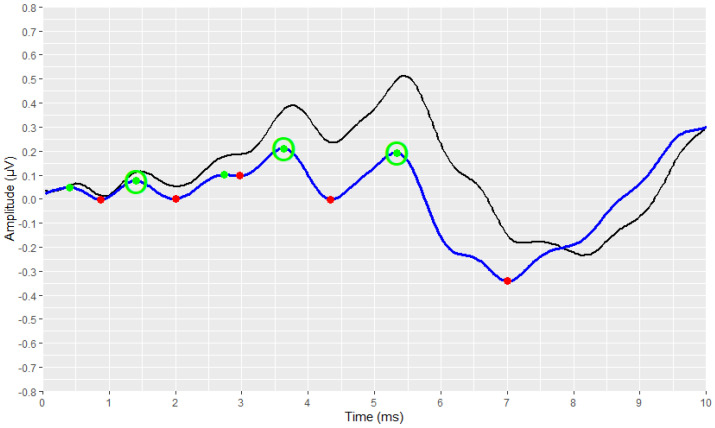
Detection of the ABR signal’s waves of interest as resulting from the implementation of the ABR automated annotation tool.

The preliminary stages of the analysis, intended to reconstruct and visualise each waveform, were successfully implemented for all signals. The outcomes of these stages are depicted in Figure 3, Figure 4, Figure 5, Figure 6, Figure 7, Figure 8, Figure 9 and Figure 10, by visualising a randomly selected waveform for each AEP subtype. At this point, it is worth noting that, regarding the clinicians’ set-up for the AMLR signals, the recording time starts 10 ms before the presentation of the tone-burst auditory stimulus. However, the AMLR signals start at time 0 and not at −10 ms. In particular, Figure 3 and Figure 4 illustrate the visualisation of the waveforms using only the raw data of the *.xml* files. 

### 3.3. Visual Display Filtering, Wave Detection and Visualisation

Subsequently, the corresponding visual display filters (presented in Table 1 and Table 2), were applied on all waveforms. Figure 5 and Figure 6 show the waveforms that were generated after the application of these filters. 

The identification and marking of all the existing peaks and troughs of the waveforms using the *find_peaks* function, are shown in Figure 7 and Figure 8. All peaks are marked with green dots, and all troughs are marked with red ones. 

Figure 9 and Figure 10 demonstrate the correct operation of the proposed automated annotation tools. In these figures, the final waveforms and waves of interest that were selected by the proposed automated tools are visualised.

**Figure 11:**
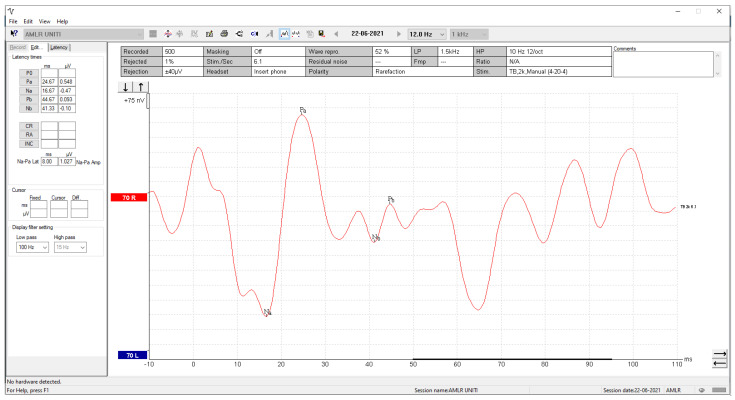
A screenshot of the selected AMLR waveform from the Interacoustics Interface.

The clinicians were asked to send us the corresponding AMLR waveform, along with their manual annotation, as it is displayed by the Eclipse software, so that we could demonstrate that both the visualisation and the automated annotation of the waves of interest were correct. By comparing the waveform from the Eclipse software (Figure 11) with the reconstructed waveform (Figure 9), we confirmed the effectiveness of the methodology followed.

### 3.4. Developed Automated Annotation Tools and Validation of Their Performances 

**Figure 12:**
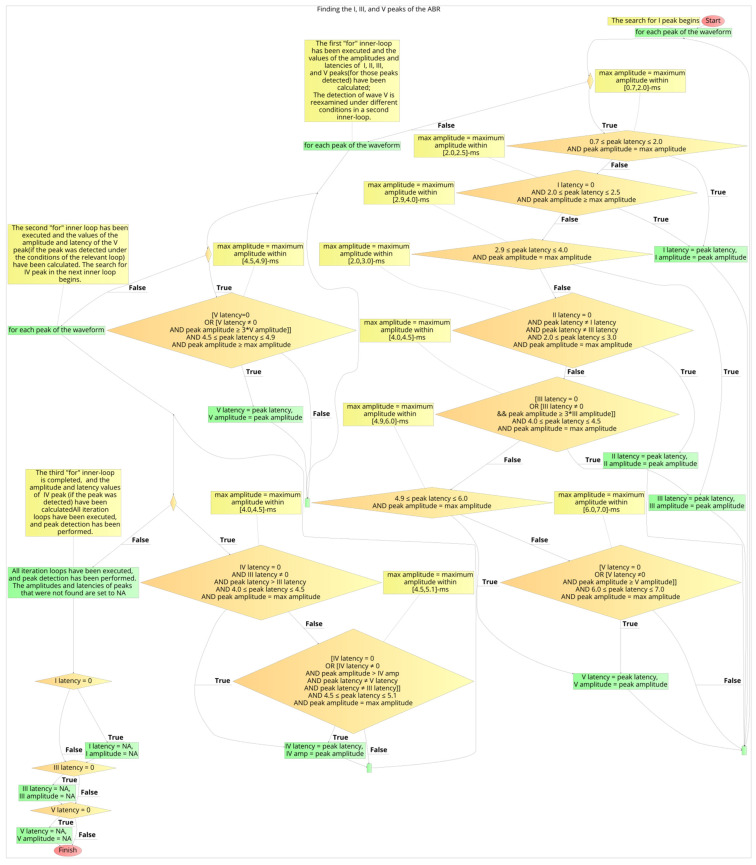
The flowchart of the automated waves detection algorithm for an ABR waveform.

**Figure 13:**
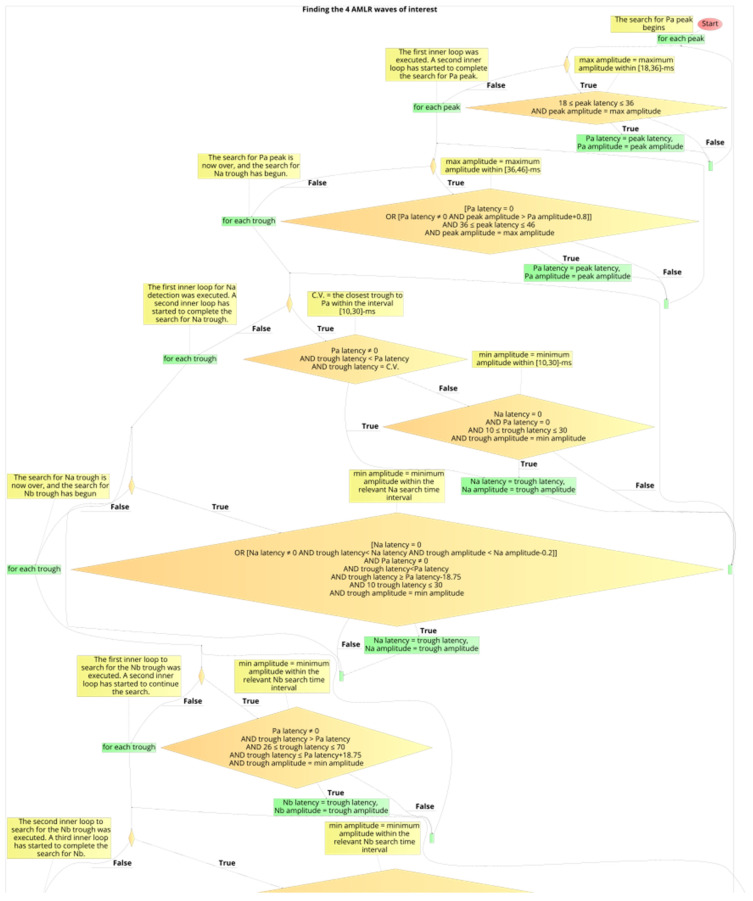

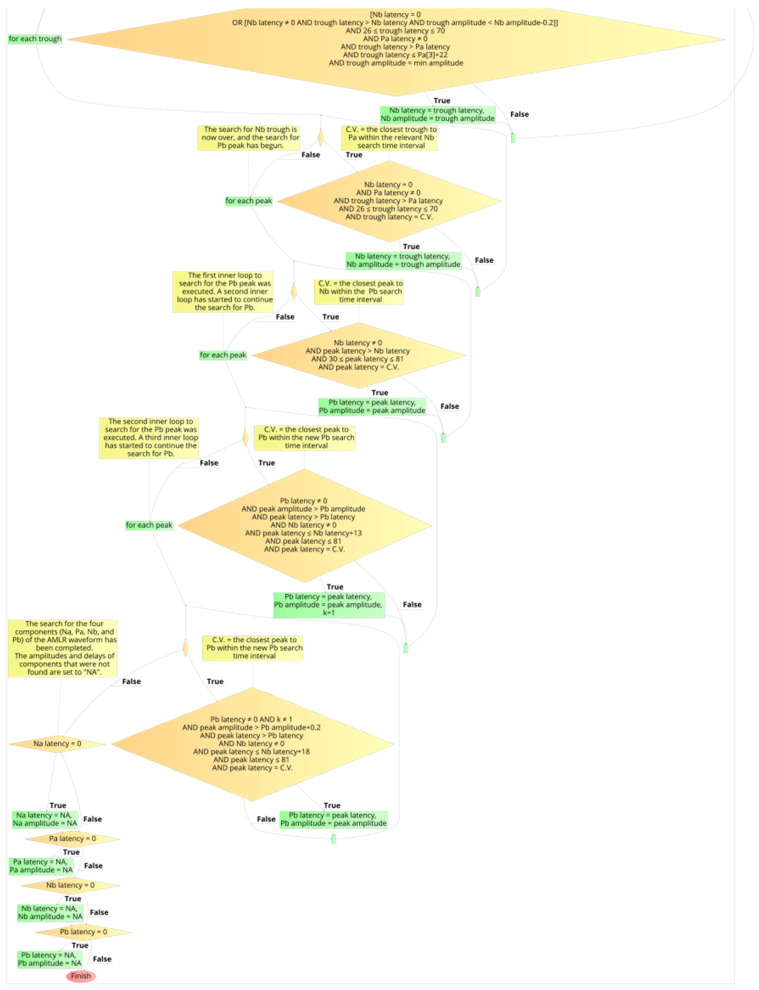
The flowchart of the automated waves detection tool of an AMLR waveform.

The development of the automated annotation tool of each AEP subtype was constituted by the four stages which were described above. After the definition of the logical expressions that resulted in the optimal performances, the developed algorithms for the ABR and the AMLR automated annotation tools were constructed. The two flow charts with the final expressions and the flow of the two annotation tools are shown in Figure 12 and Figure 13.

In order to evaluate the performance of the two proposed annotation tools, we utilised the latencies of the signals’ waves of interest. In particular, the assessment was performed based on the match rates of the tools’ proposed latencies compared with the clinicians’ selected latencies, with the latter considered as the “gold standard” annotations for our analysis.

#### 3.4.1. Validation of the ABR Automated Annotation Tool

The automated detection-tool for defining peaks I, III, and V of the ABR waveforms was tested for its match rates on a set of 698 annotated waveforms. 

In total, 359 waveforms were acquired with an acoustic stimulus of 80 dB intensity, and 339 were acquired with an acoustic stimulus of 90 dB. The match rates (accuracy) of the ABR automated tool compared with the clinicians’ annotated values for both of these signals, are presented in Table 3.

#### 3.4.2. Validation of the AMLR Automated Annotation Tool for All Waveforms

The match rates of the automated AMLR wave detection tool compared with the annotated values of the clinicians, were obtained from a set of 348 available AMLR waveforms. The match rates, with respect to the annotation of the clinicians, were:

- 91.89% for Na trough;
- 90.77% for Pa peak;
- 79.90% for Nb trough;
- 76.99% for Pb peak.

#### 3.4.3. Validation of the AMLR Automated Annotation Tool for the PAM–Absence Waveforms 

**Figure 14:**
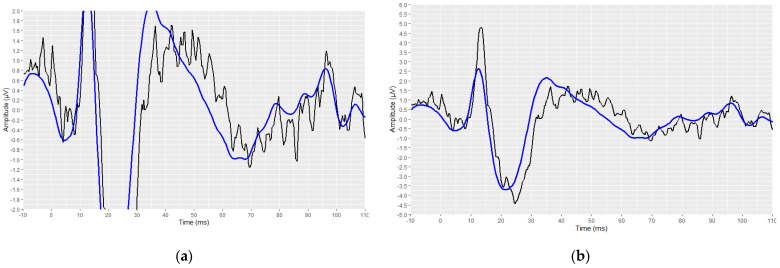
An illustration of a waveform in which a PAM artefact has been recorded: (**a**) the scale that has been used on the *y*-axis for the AMLRs plotting is also applied here; (**b**) visualization of the same waveform with a modified scale on the *y*-axis, to demonstrate the entire signal (raw signal is depicted in black and filtered signal is depicted in blue).

In the context of a more appropriate performance evaluation of the proposed AMLR annotation tool, we considered it necessary to conduct an evaluation on the annotated waveforms, this time without the inclusion of the waveforms with the PAM artefact. All available AMLR waveforms were inspected for the possible presence of PAM artefacts. From a total of 348 AMLR waveforms, 36 were found to contain a PAM artefact, and they were consequently removed. The performance-evaluation step was repeated on the 312 remaining waveforms. Figure 14 illustrates an AMLR waveform in the presence of a PAM artefact, as visualised by the RStudio interface. Figure 14a depicts the waveform using the same scales, on the coordinate axes used to depict all AMLR waveforms. The significantly higher waveform-amplitude caused by the PAM artefact prevents the complete signal from being displayed within the designated imaging frame. Figure 14b illustrates the same waveform. with the *y*-axis scale adjusted. In this manner, the complete captured signal is displayed.

The match rates compared to the annotation of the clinicians were:

- A total of 93.21% for the Na trough;
- A total of 92.25% for the Pa peak;
- A total of 83.35% for the Nb trough;
- A total of 79.27% for the Pb peak.

All ABR and AMLR waveforms for which the suggested values from the automated tools did not match the values chosen by the clinicians, were saved in two Excel *(.xlsx)* files, and reviewed by the clinicians. During the final inspection, it was observed that in the vast majority of the “failures”, the disagreement was either in waveforms that derived from “bad” recordings or in waveforms appertaining to the “grey-zone”, i.e., waveforms where there was a disagreement even among the clinicians regarding the selected values for the waves of interest. Unanimous disagreement was found in a negligible proportion of cases.

## 4. Discussion 

In this study, two automated annotation tools for ABR and AMLR signals were developed. The objective of these tools was to detect, compute, and save the latencies and amplitudes of the waves of interest for each subtype of AEP. The two tools were constructed using a four-stage implementation process. The performance of each tool was determined by the degree to which their estimated latency-values matched the clinicians’ selected values. Given the dearth of comparable algorithms and software for this purpose, the match rates were remarkably high. 

The determination of “normative” values proved to be a difficult task, as little information was available from scientifically documented sources, and disagreements or uncertainties were found regarding the proposed intervals [5,36,37]. More specifically, there was consistency between the literature sources regarding the time of occurrence of the ABRs waves of interest, but this was not the case for the AMLRs waves of interest, where inconsistencies on the proposed time intervals were found. The discrepancies in the normative values are probably due to the different stimuli and acquisition parameters used during the recording of AEPs. Moreover, the individual’s age and gender may possibly have influenced the recorded signal. In addition, based on the available waveforms, it appears that the time ranges may vary considerably among individuals (and even between the ears of the same individual), and, at the same time, are influenced by hearing loss. For example, an ABR wave after 7 ms cannot be typically considered as a peak V. However, a peak V can occur at 6.8 ms if certain conditions are met (for example, if a typical waveform is generated, its amplitude is close to the general average-amplitude for the peak V, and the distance between peaks I and III is as expected).

The visual display filters we added were reasonably and inevitably different compared with those used by the Interacoustics software, since they were derived from different operating systems and programming languages. If our developed algorithms were applied to the exact same waveforms as those derived from the Eclipse software, we believe that the match rates would be higher and perfectly objective. This conclusion applies to both the ABR and the AMLR signals.

Regarding the tool for the ABRs, the choice of the intervals appeared to be precise. It was observed that, in some cases, with the application of the selected visual-display-filters, the signal was minimally altered, compared with the one inspected by clinicians using the Eclipse software. Therefore, some peaks, mainly peak V and peak I, were not present, and thus not detected by our tool, and the match rate for these peaks was lower than that for the other wave. Furthermore, it was observed that for some mis-annotated waveforms, the fault was with the clinicians, who probably performed the annotation without the addition of the visual display filters, which were indicated in accordance with the pre-defined set up. Taking these two factors into account, the match rates were estimated to be even higher.

Regarding the AMLR tool, there was a lower match-rate, especially for the Nb and Pb wave-latencies. However, for these cases there were differences even between the clinicians’ annotation for the selection of the waves of interest. This indicates that our “gold standard”, which was used to develop and evaluate the AMLR automated annotation tool, is not completely objective. During an overall inspection, there was a trend towards a lower confidence-level on the part of the clinicians for the majority of waves that were not correctly annotated. This poor confidence-level was translated as an inconsistency among the clinicians’ annotations for these waves. Hence, for these cases, the clinically annotated waveforms used as a reference were randomly selected. This could mean that some mis-matched selections of our tool were randomly assigned as false choices, or vice versa. The presence of these “grey-zone” waveforms inevitably negatively affected the performance of the AMLR automation annotated tool.

**Figure 15:**
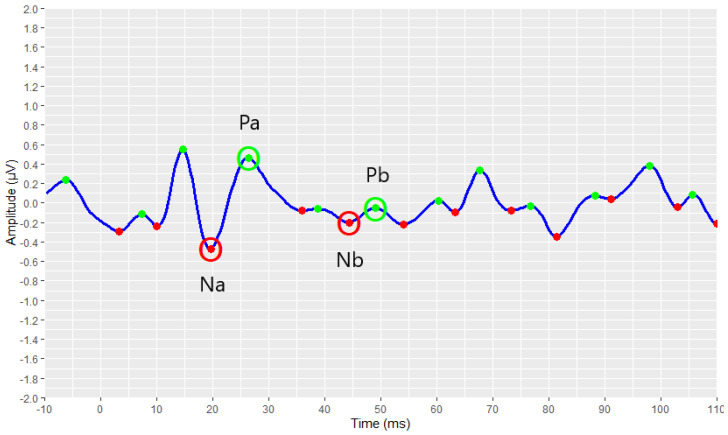
Selecting the waves of interest in a waveform where it is not easy to detect them.

Additionally, it is worth mentioning that the AMLR tool detected waves of interest in some waveforms where a wave distinction was not obvious. Such an example is illustrated in Figure 15. A simple observer or a clinician without appropriate experience could incorrectly select the peak that is close to 15 ms as the Pa peak, based on the fact that the Pa peak is usually the one with the largest amplitude value in the signal. In addition to being incorrect, this choice could lead to the incorrect selection of the three remaining waves of interest. In the present waveform, the Pa peak is located near 27 ms, as correctly selected by the AMLR automated annotation tool, as the value of 15 ms is not included in the defined time-interval for the detection of the Pa wave. Therefore, it is evident that the definition of the proper time-interval for the detection of each wave is crucial for properly selecting the waves of interest.

The choice of setup for both the stimulus (e.g., the type of stimulus and its intensity) and the acquisition parameters (e.g., the sample rate and the filters used), directly affects the AEP signals. The careful manipulation of stimulus parameters, based on research findings and clinical experience, plays a critical role on the recording quality. The two tools were created from data (waveforms) recorded and extracted from the Eclipse system (module EP25) of Interacoustics, and exactly the same stimulus settings and parameters were used for the recordings for each test. The settings used were those suggested by the clinicians of the UNITI project consortium as being the most appropriate. The application of these automated tools to signals derived from different settings (setup) may not perform as well, given the altered parameters of the received signals.

At this juncture, we deemed it useful to mention some previously conducted studies aimed at performing automated annotation of ABR signals [40,41,42]. In the earliest study identified [40], ABR signals were evaluated using dynamic time warping. Dynamic time warping is a method in which a temporal sequence of values is stretched or contracted, to resemble another sequence. The generated warp-average mimics the waveform of a typical subject more closely than the conventional average does. Hence, each individual’s waveform was compared to a normal template, and in this way, warping could identify the peaks that corresponded most closely to the peaks in the normal template. Although this method can be useful in identifying the waves in normal ABR waveforms, it is not accurate in identifying the waves in abnormal waveforms. In another study [41], the fitted-parametric-peaks (FPP) method provided an automatic evaluation of the quality of ABR signals and parameterized waves III and V, in terms of amplitude, latency, and width. This method was based on the use of synthesised peaks that were adjusted to the ABR response. The study mentioned that the FPP method was tested and evaluated on waveforms from a relatively small number of individuals (*n* = 18), all of whom had normal hearing. In addition, no attempt was made to identify I-waves, which are equally useful for the evaluation of ABR signals. The most recent study [42] proposed an automated procedure for extracting individual ABR wave-latencies and amplitudes, based on a well-established non-linear-curve-registration methodology. The implementation procedure involved the pre-processing of individual ABRs and the alignment of the responses, using continuous-monotone time-warping, which carried the danger of over-alignment and therefore was applied with particular care. The waves I and V latencies and amplitudes were retrieved from an ABR data-set consisting of 23 normal healthy subjects and 12 different experimental settings. The values of these were used to assess the study’s approach.

The performance comparison of the automated tool for ABR waves detection of the present study with the other tools developed for the same purpose, cannot be global. The reasons are the following: (i) the waveforms on which the performances were evaluated were derived from different software, (ii) the clinicians used different setups for capturing their recordings, and (iii) the recordings were retrieved by different subjects. On top of this, we consider some major superiorities of our tool. Firstly, the tool can detect all the waves of interest in the ABR signals that clinicians use for signal interpretation, rather than a subset of them. Secondly, the waveforms on which our tool was tested came from subjects who overwhelmingly were hearing-impaired. AEPs, in the broad sense, are hearing-testing methods. Evaluating the performance of equivalent tools by including waveforms exclusively from healthy people with normal hearing is easier, and leads to better performance results, as these signals are more likely to fall into “normal” ranges. However, in these cases, performance rates cannot be universal indicators of tool performance. Lastly, its performance was evaluated on waveforms from a considerably larger dataset of subjects, compared with the other studies.

Regarding the AMLR waveforms, no individual attempts at achieving automated-annotation were identified, as was also mentioned in the Introduction. Additionally, for this type of AEP, the Eclipse software does not suggest values for the waves of interest of these signals. Therefore, the proposed AMLR tool in this study is the first attempt towards an automated annotation tool for these signals, to the best of our knowledge.

Despite the high-performance results of the two proposed tools, a number of limitations were identified, which are assessed as having significantly influenced the tools’ development and performances. The most important ones are outlined hereinafter. A major limitation of our analysis was the fact that the results of our automated annotation tools were not compared with other automated-annotation-software results, as these data were not available. However, to the extent of our knowledge, the current software’s annotations are not satisfactory for the clinicians. Moreover, the lack of knowledge of accurate visual-display-filters or, ideally, the non-integration of the tools into the software system from which the signals were derived, was another limitation. The lack of universally accepted “normative” time-intervals for the occurrence of the waves of interest was an equally important limitation for the proper determination of extended time-intervals for wave detection. The presence of “bad” recordings, and, in the case of AMLR waveforms, the presence of “grey-zone” waveforms, were inevitable obstacles to finding the waves of interest. Considering all of the above, we can conclude that the creation of the AMLR automated annotation tool proved to be a more difficult task. Combating the above limitations will substantially assist in optimising these tools. The creation of guidelines and protocols for the recording of “high-quality” signals (i.e., the proper set-up for the recording of each test, as well as the details relating to the instructions to be given to the examinees before and during these tests), and the inclusion of commonly accepted normative-intervals for the waves of interest, will benefit both the development and optimization of automated tools and the clinicians themselves, for a better interpretation and diagnosis of these signals.

## 5. Conclusions

Overall, the clinical evaluation of AEPs focuses on the latencies and amplitudes of the waves of interest. Therefore, a prerequisite for the computation of these metrics is the detection of these waves. The process of wave detection has proven to be a complex procedure. This is evidenced by the fact that the existing software for this purpose is of little use in practice, and, therefore, this procedure is mostly fulfilled by the manual annotation of the clinicians. The ultimate aim of the study was to address this difficulty by developing two automated annotation tools, one for the ABR- and one for the AMLR-test. The application of these tools led to a direct and automated determination of the latencies and amplitudes of the waves of interest. The match rates of the two tools were very encouraging, indicating the removal of the limitations reflected in the commercial devices and their proprietary software. We suggest the adoption of such tools for AEP signal analysis, as their functionality appears to be highly helpful to clinicians, audiology physicians, and researchers interested in the direct interpretation of these signals. Future investigations are therefore deemed more than necessary to validate the kind of conclusions drawn from this study.

## Figures and Tables

**Table 1:** Stimulus and acquisition parameters for ABR recordings.

Stimulus Parameters		Acquisition Parameters	
Type of transducer	Insert phone	Analysis time	15 ms
Sample Rate	30 kHz	Sweeps	4000
Type of stimulus	Click	Mode	Monaural
Polarity	Alternate	Electrode montage	Vertical (Fpz, Cz, M1/M2)
Repetition rate	Stimuli per second: 22 Hz	Filter setting for input amplifier	Low Pass: 1500 Hz; High Pass: 33 Hz 6 dB/octave
Intensity	80 dB nHL; 90 dB nHL	Preliminary display settings—Visual display filters	Low pass: 1500 Hz; High Pass: 150 Hz
Masking	Off		

**Table 2:** Stimulus and acquisition parameters for AMLR recordings.

Stimulus Parameters		Acquisition Parameters	
Type of transducer	Insert phone	Analysis time	150 ms
Sample Rate	3 kHz	Sweeps	500
Type of stimulus	2 kHz Tone Burst	Mode	Monaural
Duration of stimulus	28 sine waves in total; Rise/fall: 4; plateau: 20	Electrode montage	Vertical (Fpz, Cz, M1/M2)
Polarity	Rarefaction	Filter setting for input amplifier	Low Pass: 1500 Hz; High Pass: 10 Hz, 12 dB/octave
Repetition rate	Stimuli per second: 6.1 Hz	Preliminary display settings—Visual display filters	Low pass: 100 Hz; High Pass: 15 Hz
Intensity	70 dB nHL		
Masking	Off		

**Table 3:** Validation of the ABR automated annotation tool.

Wave of Interest	ABR 80 dB	ABR 90 dB
Peak I	94.88%	93.86%
Peak III	98.51%	98.85%
Peak V	92.97%	91.51%

## Data Availability

The data presented in this study are available on reasonable request from the corresponding author.
